# Acriflavine targets oncogenic STAT5 signaling in myeloid leukemia cells

**DOI:** 10.1111/jcmm.15612

**Published:** 2020-07-15

**Authors:** Rawan Hallal, Rawan Nehme, Marie Brachet‐Botineau, Ali Nehme, Hassan Dakik, Margaux Deynoux, Persio Dello Sbarba, Yves Levern, Kazem Zibara, Fabrice Gouilleux, Frédéric Mazurier

**Affiliations:** ^1^ Université de Tours EA7501 GICC Tours France; ^2^ CNRS ERL7001 LNOx Tours France; ^3^ PRASE Lebanese University Beirut Lebanon; ^4^ Dipartimento di Scienze Biomediche Sperimentali e Cliniche “Mario Serio” Università degli Studi di Firenze Florence Italy; ^5^ INRAE Imagerie en Infectiologie UMR Infectiologie et Santé Publique Université de Tours Nouzilly France; ^6^ Biology Department Faculty of Sciences‐I Lebanese University Beirut Lebanon

**Keywords:** acriflavine, apoptosis, cell cycle, chronic myeloid leukaemia, resistance, STAT5

## Abstract

Acriflavine (ACF) is an antiseptic with anticancer properties, blocking the growth of solid and haematopoietic tumour cells. Moreover, this compound has been also shown to overcome the resistance of cancer cells to chemotherapeutic agents. ACF has been shown to target hypoxia‐inducible factors (HIFs) activity, which are key effectors of hypoxia‐mediated chemoresistance. In this study, we showed that ACF inhibits the growth and survival of chronic myeloid leukaemia (CML) and acute myeloid leukaemia (AML) cell lines in normoxic conditions. We further demonstrated that ACF down‐regulates STAT5 expression in CML and AML cells but activates STAT3 in CML cells in a HIF‐independent manner. In addition, we demonstrated that ACF suppresses the resistance of CML cells to tyrosine kinase inhibitors, such as imatinib. Our data suggest that the dual effect of ACF might be exploited to eradicate de novo or acquired resistance of myeloid leukaemia cells to chemotherapy.

## 1 INTRODUCTION

Acriflavine (ACF), a mixture of 3,6‐diamino‐10‐methylacridinium chloride (trypaflavine) and 3,6‐diaminoacridine (proflavine), was first described in 1912 by the Nobel prize Paul Ehrlich as an antiseptic and has been since used to kill parasites. US Food and Drug Administration (FDA) has also approved ACF for human topical (non‐oncological) uses in wound healing. In addition, owing to its intercalating properties, ACF has been extensively studied as a fluorescent molecule in detecting bacteria.^1^ More recently, high‐throughput drug screening identified ACF as a potent antitumoral molecule in colorectal cancers (CRC).^2^ To date, this effect has been confirmed in various solid tumours such as osteosarcoma, breast, brain, lung, hepatic and pancreatic cancers.^3^, ^4^, ^5^, ^6^, ^7^ Moreover, ACF has also increased the cytotoxic effects of chemotherapeutic compounds such as 5‐fluorouracil (5‐FU) or melphalan in cancer cells.^8^, ^9^ Other studies have demonstrated that ACF effectively inhibits drug resistance and epithelial‐mesenchymal transition (EMT) of pancreatic and hepatic cancer cells.^7^ Importantly, long‐term administration of ACF to AIDS patients, as an antiviral agent, has not revealed any major side effects suggesting that it could be employed alone or in combination with other drugs to overcome chemotherapy resistance of cancer cells.^10^

Mechanistic studies have identified DNA‐dependent protein kinases C (DNA‐PKcs) and topoisomerase I and II as direct targets of ACF in various cancer cells.^2^, ^9^ ACF can also enhance P53 activation in γ‐irradiated colorectal cancer cells and, consequently, potentiate radiation‐induced cell death.^11^ Nowadays, ACF is frequently used as an inhibitor of HIF dimerization and/or protein expression.^12^ HIFs are main transcription factors that actively promote the progression of a variety of solid tumours as well as leukaemias.^13^, ^14^, ^15^ For instance, HIF‐1α has been reported as a crucial driver of chronic myeloid leukaemia (CML) development induced by the BCR‐ABL oncogene. Indeed, in the hypoxic bone marrow (BM) microenvironment, HIF‐1α has been found to support the persistence of CML leukaemic stem cells (LSCs) in a BCR‐ABL kinase–independent manner.^16^ HIF‐1α‐induced metabolic reprogramming is also required for imatinib (IM) resistance of CML cells associated with BCR‐ABL up‐regulation.^17^ Cheloni et al have recently shown that ACF inhibits the growth and survival of CML cells as well as the stem cell potential of CML LSCs, while sparing normal haematopoietic stem/progenitor cells. They further demonstrated that ACF blocks leukaemia development and reduced CML LSCs maintenance in a CML mouse model.^18^ Based on these studies, ACF has been proposed as a new therapeutic approach to prevent the relapse of CML.

Although HIF proteins are mainly activated by hypoxia, several reports indicated that expression and/or activity of HIF‐1α/HIF‐2α are also regulated by STAT3 (Signal Transducer and Activator of Transcription 3) and STAT5 in normal and cancer cell types including CML LSCs.^19^, ^20^, ^21^ STAT3 and the two closely related STAT5A and STAT5B proteins are key players in the development of solid and haematopoietic cancers.^22^, ^23^, ^24^ STAT3 and STAT5 are aberrantly tyrosine‐phosphorylated in cancer cells and are crucial effectors of different tyrosine kinase oncogenes (TKO) such as FLT3‐ITD in acute myeloid leukaemia (AML) and BCR‐ABL in CML.^25^, ^26^ Using mouse models of CML, previous studies have demonstrated that, while STAT3 and STAT5 are both necessary for initiation of the disease, only STAT5 is required for the maintenance of leukaemia.^27^ In addition, reports have indicated that STAT3 and STAT5 contribute to the resistance of CML cells to tyrosine kinase inhibitors (TKI).^28^, ^29^ STAT3 and STAT5 are now well‐recognized therapeutic targets in haematologic malignancies and have been the subject of intense investigations for the development of selective pharmacological inhibitors.^30^ Some of these inhibitors have been identified by high‐throughput screening of drug repurposing libraries. For instance, the antiparasitic pyrimethamine or the antipsychotic pimozide have been shown to, respectively, inhibit the phosphorylation of STAT3 and STAT5 in different haematopoietic cancers, whereas the antidiabetic drug pioglitazone was found to reduce *STAT5A* and *STAT5B* gene expression in CML cells.^21^, ^31^, ^32^ In all cases, these drugs whether used alone or in combination therapies decreased the growth and survival of solid tumours or leukaemic cells, and resensitized resistant cancer cells to chemotherapy.

Acriflavine effectively inhibits tumour cell growth in both hypoxic and normoxic conditions. Since ACF hampered CML cells development and IM resistance, we investigated whether ACF could regulate STAT signalling in leukaemic cells. Here, we demonstrated that STAT3/5A/5B expression and/or activation were strongly impacted by ACF in CML and AML cells. Thus, ACF‐mediated modulation of STAT3/5 signalling might also be responsible for the antileukaemic activity of this compound.

## 2 MATERIAL AND METHODS

### 2.1 Cell cultures and reagents

K‐562, KU‐812, KCL‐22 and MV‐4‐11 cell lines were obtained from Deutsche Sammlung von Mikroorganismens und Zellkulturen (DSMZ), and IM‐sensitive (K562S) and IM‐resistant (K562R) BCR‐ABL^+^ cells from American Type Culture Collection (ATCC). Cells were maintained according to the supplier's recommendations. All cell lines were cultured in RPMI 1640 medium containing 2 mmol/L glutamine, 100 U/mL penicillin, 100 µg/mL streptomycin (Gibco, Thermo Fisher, Waltham, MA, USA) and 10% foetal bovine serum (FBS, Thermo Scientific, Sigma‐Aldrich, St Louis, MO, USA) at 37°C, 5% CO_2_. K562R cells were cultured with 1 µmol/L imatinib mesylate (IM). IM was purchased from Selleckchem (Houston, TX, USA), Acriflavine hydrochloride and proflavine hydrochloride from Sigma‐Aldrich (St‐Louis, MO, USA). Cells were cultured at 1% O_2_ (Xvivo System, Biospherix, Parish, NY, USA).

### 2.2 Cell proliferation assays

Cells were washed and resuspended in culture medium at a concentration of 2 × 10^5^ cells/mL. Then, they were dispensed into 96‐well culture microplates (Falcon^®^) containing 10 μL/well of serial drug dilutions of ACF. PBS alone was used as control. After treatment at desired times, 10 μL of a 5 mg/mL solution of MTT was added to each well. Plates were then incubated for another 4 hours before the addition of 10% sodium dodecyl sulphate (SDS) solution with 0.01 M hydrochloric acid to solubilize the formazan crystals. After an overnight incubation at 37°C, the absorbance was measured at λ = 590 nm using a spectrophotometer (CLARIOstar^®^ Monochromator Microplate Reader; BMG Labtech, Offenburg, Germany). Living cells were also counted using the trypan blue dye exclusion method.

### 2.3 RNA extraction and real‐time reverse‐transcription quantitative PCR (RT‐qPCR)

Total cellular RNA was extracted by TRIzol (Invitrogen) and quantified using NanoDrop Lite spectrophotometer. Five micrograms of RNA were reverse‐transcribed using the SuperScript^®^VILO TM cDNA synthesis kit (Invitrogen, Paris, France) as recommended by the supplier. The resulting cDNAs were used for real‐time quantitative PCR (RT‐qPCR). PCR primers (*PIM1*: *for 5′‐*TTTCGAGCATGACGAAGAGA‐3′, *rev* 5′*‐*GGGCCAAGCACCATCTAAT‐3′; *CISH:* 5′‐ AGCCAAGACCTTCTCCTACCTT‐3′, *rev* 5′‐TGGCATCTTCTGCAGGTGT‐3′; *STAT5A*: *for* 5′‐TCCCTATAACATGTACCCACA‐3′, *rev* 5′‐ATGGTCTCATCCAGGTCGAA‐3′; *STAT5B*: *for* 5′‐TGAAGGCCACCATCATCAG‐3′, *rev* 5′‐TGTTCAAGATCTCGCCACTG‐3′, *cMYC*: *for* 5′‐GCTGCTTAGACGTGGATTT‐3′, *rev* 5′‐TAACGTTGAGGGGCATCG‐3′, *STAT3*: *for* 5′‐CTCTGCCGGAGAAACAGG‐3′, *rev* 5′‐CTGTCACTAGAGCTGATGGAG‐3′, *GAPDH: for* 5′‐AGCCACATCGCTCAGACAC‐3′, *rev* 5′‐GCCCAATACGACCAAATCC‐3′, *ACTB: for* 5′‐ATTGGCAATGAGCGGTTC‐3′, *rev* 5′‐CGTGGATGCCACAGGACT‐3′, *YWHAZ: for* 5′‐GCAATTACTGAGAGACAACTTGACA‐3′, *rev* 5′‐TGGAAGGCCGGTTAATTTT‐3′, *RPL13A: for* 5′‐CAAGCGGATGAACACCAAC‐3′, *rev* 5′‐TGTGGGGCAGCATACCTC‐3′) were designed with the ProbeFinder software (Roche Applied Sciences, Basel, Switzerland) and used to amplify the RT‐generated cDNAs. Primers validation was done on Stratagene cDNA mix (Agilent Technologies). qRT‐PCR analyses were performed on the Light Cycler 480 thermocycler II (Roche). *GAPDH* (glyceraldehyde‐3‐phosphate dehydrogenase), *ACTB* (actin beta), *YWHAZ* and *RPL13A* were used as reference genes for normalization of RT‐qPCR experiments. Each reaction condition was performed in triplicate. Relative gene expression was analysed using the 2^−ΔΔC^ *^t^* method.^33^

### 2.4 Apoptosis and cell cycle analysis

Cells were cultured in the absence or presence of different concentrations of ACF. Three days after, cells were harvested and washed with cold PBS, stained (10^6^ cells) in a buffer containing APC (Allophycocyanin)‐annexin V (BioLegend) for 15 to 30 min at room temperature in the dark and analysed with an Accuri™ C6 flow cytometer (Becton‐Dickinson, Le Pont de Claix, France).

For cell cycle analysis, cells were incubated with ethanol 100% for 1 hours at 4°C and then washed with a PFT permeabilization solution containing PBS 1x, SVF 1% and 0.25% Triton 100x. Cells were then stained for 30 minutes at room temperature with anti‐Ki67‐Alexa Fluor 647 monoclonal antibody, with 7‐amino‐actinomycin D (7‐AAD) or the corresponding isotype as control (BD Biosciences, Le Pont de Claix, France). Cells were then acquired by flow cytometry (MoFlo Astrios EQ; Beckman Coulter, Villepinte, France). Data were analysed with The FlowJo^®^ software V10.1 (Tree Star Inc).

### 2.5 Western blotting analysis

Cells were suspended in Laemmli buffer (62.5 mmol/L Tris‐HCl pH 6.8, 2% SDS, 10% glycerol, 5% β‐mercaptoethanol, 0.005% bromophenol blue). Extracted proteins were heated at 95°C for 5 minutes. A volume of protein corresponding to 5 × 10^5^ cells were then loaded per well in a 4%‐15% gradient polyacrylamide gel (Bio‐Rad, Marnes‐la‐Coquette, France) and separated by SDS‐polyacrylamide gel electrophoresis (PAGE). Proteins were then transferred onto 0.2 µm nitrocellulose membranes (Bio‐Rad). Membranes were blocked for 1 hour at room temperature in TBS‐Tween (0.2%) buffer containing 5% milk and incubated overnight at 4°C with the following antibodies: Caspase 3 (9662), cleaved Caspase 3 (9661), P21^Waf1^ (2947), P27^kip1^ (2552), PARP (9532), CDK4 (12790), Bcl‐X_L_ (2764), γH2AX (2577), P‐Y^245^‐c‐Abl (2861), c‐Abl (2862), P‐Y^705^‐STAT3 (9131), P‐Y^694/699^‐STAT5 (4322), P‐T^308^‐AKT (13038), AKT (pan) (4685), Actin (4967) (Cell Signaling Technology, Danvers, MA, USA), STAT3 (610190) and STAT5 (610192) (BD Transduction Laboratories, Franklin Lakes, NJ, USA), STAT5A (13.3600) and STAT5B (13.5300) (Zymed/Thermo Fisher Scientific, Waltham, MA, USA), and HIF‐2α (ab199) (Abcam, Cambridge, UK). Membranes were developed with the ECL chemiluminescence detection system (GE Healthcare, Velizy‐Villacoublay, France) using horse radish peroxidase (HRP)‐conjugated anti‐rabbit or anti‐mouse antibodies (Vector labs, Peterborough, UK).

### 2.6 Statistical analyses

Results are expressed as mean ± SD for 3‐6 independent experiments. Statistical significance of differences was determined using two‐way ANOVA test for Apoptosis and RT‐qPCR analysis, whereas multiple *t* test was used for cell cycle analysis. The correction of multiple comparisons was done using the Holm‐Sidak's method. GraphPad Prism (version 6.01; San Diego, CA, USA) software and R software were used to perform the analysis. The *P* value were determined, and values <0.05, <0.001 and <0.0001 (*, **, ***, respectively) were considered significant.

## 3 RESULTS

### 3.1 ACF inhibits growth and survival of K562 cells

**FIGURE 1:**
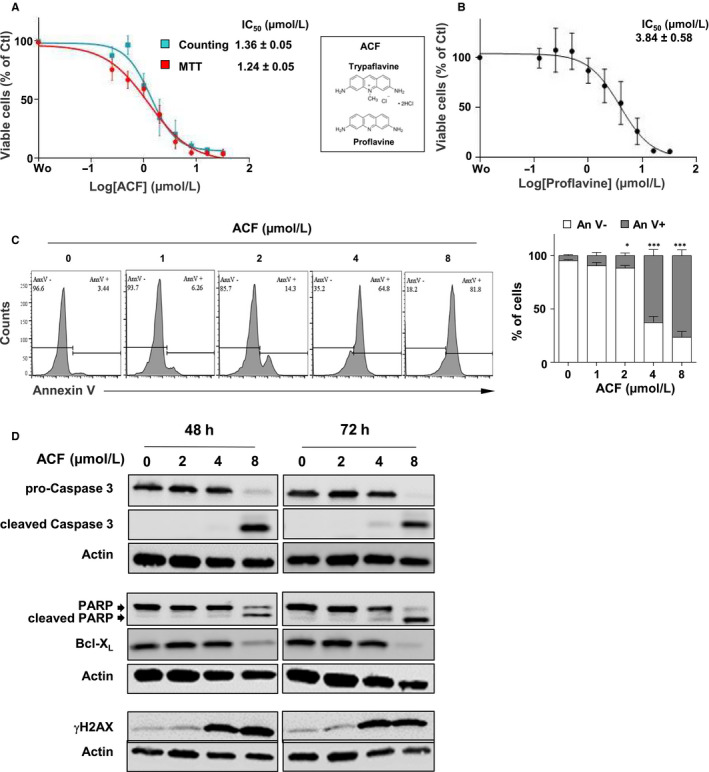
Effect of acriflavine (ACF) on growth and survival of K562 cells. A, K562 cells were treated with various concentrations of ACF or not (PBS) for 72 h. Cell viability was determined by MTT and trypan blue dye exclusion assays (data are presented as mean ± SD of five independent experiments done in triplicates). IC_50_ values are indicated. B, Cells were treated with various concentrations of proflavine, and the percentages of viable cells were determined by MTT assays (data are presented as mean ± SD of three independent experiments done in triplicates). Chemical structures of proflavine and trypaflavine are shown. C, Cells cultured without (PBS) or with increasing concentrations of ACF for 72 h were stained with AnnexinV coupled with APC to determine the percentages of apoptotic cells by flow cytometry. One representative experiment is shown (left panel). Data are presented as mean ± SD of six independent experiments done in triplicates. Two‐way ANOVA followed by Holm‐Sidak's multiple comparison test was used to examine the significance of ACF treatment on apoptosis (*P < 0.05; ***P < 0.0001). D, Protein extracts from K562 cells cultured for 48 h or 72 h with increasing concentrations of ACF were analysed by western blot with the indicated antibodies (n = 3). Actin served as the loading control

We first investigated the growth inhibitory effects of ACF on CML cells in normoxic conditions. For this purpose, a CML cell line, K562, was treated with various ACF concentrations. Results showed that ACF reduced CML cell growth and viability, using trypan blue dye exclusion and MTT assays. ACF at a concentration of ~1.3 µmol/L reduced K562 cell viability by 50% (*P* < 0.05), whereas concentrations above 4 µmol/L caused more than 90% decrease (Figure 1A). ACF is a mixture of trypaflavine and proflavine with a ratio of 2:1. Proflavine is required to maintain the stability of ACF. To examine its contribution to the inhibitory effect of ACF, K562 cells were incubated with increasing concentrations of proflavine (Figure 1B). Results showed that proflavine was three times less efficient than ACF in inhibiting CML cell growth (Figure 1A vs Figure 1B), indicating that, although both proflavine and trypaflavine are active compound, the latter exhibited a significantly higher toxicity.

In order to determine whether the reduced number of cells is due to ACF‐mediated cell death, K562 cells were incubated with ACF for 72 hours prior to Annexin V staining for the detection of apoptotic and necrotic cells. Cell death was strongly induced after treatment with 4 μmol/L ACF (~75%, *P* < 0.001) but remained low with doses below 2 µmol/L **(**Figure 1C **)**. To validate these results, the effects of ACF on markers of apoptosis were determined by western µmol/L ACF, while both caspase 3 and PARP were fully cleaved with 8 μmol/L ACF **(**Figure 1D **)**. The induction of apoptosis was also confirmed by the ACF‐mediated down‐regulation of the anti‐apoptotic protein Bcl‐X_L_. ACF is known as an intercalating agent that probably creates DNA damage, thereby triggering apoptosis.^34^ We thus analysed the impact of ACF on the phosphorylation of histone H2AX, a marker of DNA damage. H2AX phosphorylation was clearly induced after treatment with 4 μmol/L ACF **(**Figure 1D), a concentration high enough to induce apoptosis in K562 cells **(**Figure 1C).

**FIGURE 2:**
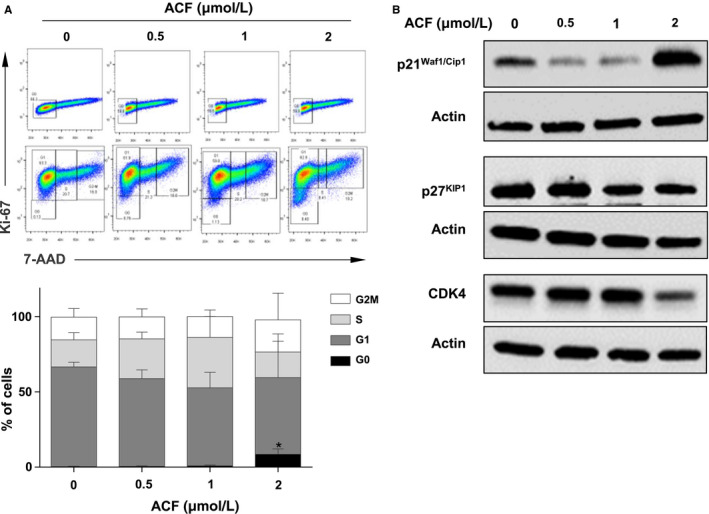
Impact of acriflavine (ACF) on the cell cycle of K562 cells. A, K562 cells treated with ACF for 72 h were stained with 7‐AAD and Alexa Fluor‐conjugated anti‐Ki67 or Alexa Fluor‐conjugated IgG isotype control. The distributions of cell cycle phases were then estimated by flow cytometry. One representative experiment is shown (upper part). Data are presented as mean ± SD of three independent experiments (lower part). B, Protein extracts from K562 cells cultured for 72 h with increasing concentrations of ACF were analysed by Western blot with the indicated antibodies (n = 3). Actin served as the loading control

Acriflavine concentrations ranging between 1 and 2 µmol/L inhibited K562 cell growth without detectable, or with minor effects, on apoptosis suggesting that low drug concentrations might affect cell cycle regulation. The distribution of K562 cells through cell cycle phases was thus determined after treatment with various ACF concentrations using 7‐AAD/Ki‐67 staining. At the 2 µmol/L concentration, ACF increased the percentage of cells arrested in the G0/G1 phase, providing evidence that ACF induced quiescence of K562 cells (Figure 2A). Consistent with these data, after treatment with 2 µmol/L ACF, we observed an activation of the cell cycle inhibitor p21^cip1/waf1^ and a reduction of the expression of cyclin‐dependent kinase 4 (CDK4) while that of the cell cycle inhibitor p27^kip1^ protein was unchanged (Figure 2B).

### 3.2 ACF regulates STAT3 and STAT5 signalling in myeloid leukaemia cells

**FIGURE 3:**
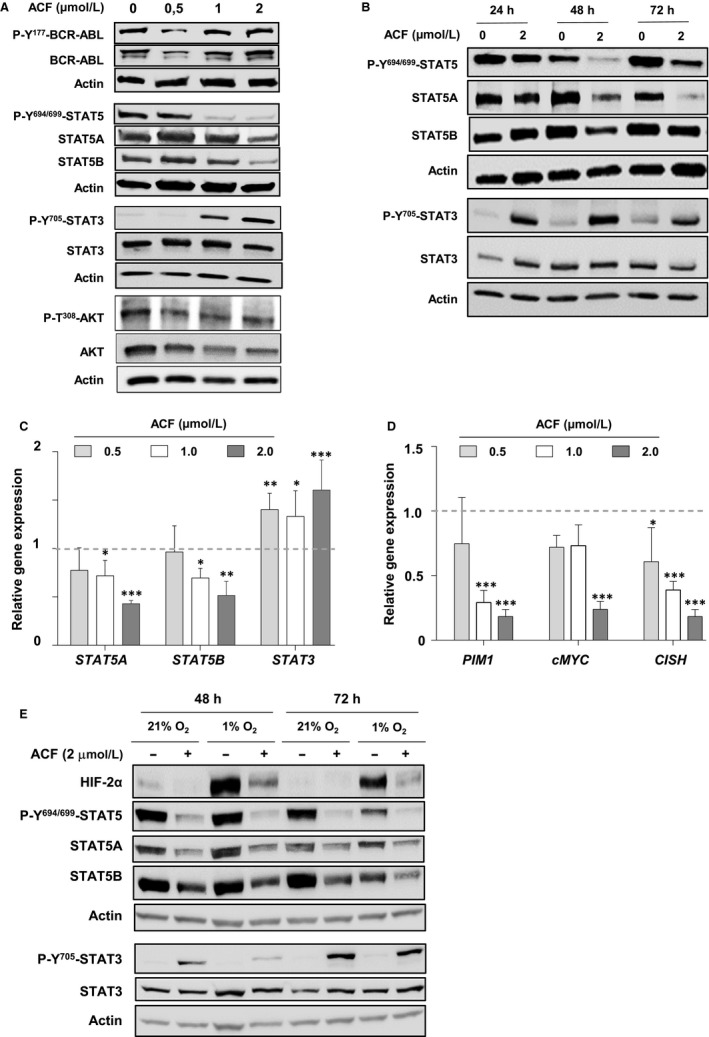
Acriflavine (ACF) targets STAT3/STAT5 signalling in K562 cells. A, Protein extracts from K562 cells cultured for 72 h without (PBS) or with increasing concentrations of ACF were analysed by western blot with the indicated antibodies (n = 3). Actin served as the loading control. B, Cells were cultured with ACF (2 µmol/L) or not (PBS) for the specified times. Protein extracts were then prepared and analysed by immunoblotting with the indicated antibodies (n = 3). Actin served as the loading control. C, qRT‐PCR analysis of *STAT5A*, *STAT5B* and *STAT3* expression in K562 cells treated or not (PBS) with increasing concentrations of ACF for 72 h. Results are presented as the fold change in *STAT3/5A/5B* gene expression in treated cells normalized to internal control genes (*GAPDH*, *ACTB*, YWHAZ and *RPL13a*) and relative to the control condition (normalized to 1) (n = 4 done in triplicates, data are mean ± SD, *P < 0.05; one‐sample *t* test). D, qRT‐PCR analysis of *PIM1*, *cMYC* and *CISH* in K562 cells treated or not with increasing concentrations of ACF for 72 h. Results are presented as the fold change in *STAT3/5* target gene expression in treated cells normalized to internal control genes (*GAPDH*, *ACTB* and *RPL13a*) and relative to the control condition (normalized to 1) (n = 4 done in triplicates, data are mean ± SD, *P < 0.05; **P < 0.001; ***P < 0.0001, two‐way ANOVA test). E, K562 cells were cultured in normoxic (21% O_2_) or hypoxic (1% O_2_) conditions and treated or not with ACF (2 µmol/L). Protein extracts were then prepared and analysed by western blot with the indicated antibodies (n = 2). Actin served as the loading control

Our data indicated that low doses of ACF might trigger cell cycle arrest by disturbing cell signalling pathways in leukaemic cells. In order to investigate this hypothesis, we first determined the phosphorylation of BCR‐ABL and its downstream effectors STAT3, STAT5 and AKT in K562 cells treated with low concentrations (≤2 µmol/L) of ACF. A dose/response effect of a 72 hours ACF treatment is reported in Figure 3A. Results showed that BCR‐ABL phosphorylation was unaffected by ACF. In sharp contrast, at the 1 µmol/L concentration, ACF strongly inhibited the tyrosine (Y^694/699^) phosphorylation of STAT5, while inducing STAT3 tyrosine (Y^705^) phosphorylation. The decrease of STAT5 phosphorylation was due to a dose‐dependent reduction of both STAT5A and STAT5B protein expression levels. However, ACF did not significantly affect AKT phosphorylation and expression suggesting that ACF selectively targets STAT3 and STAT5 downstream of BCR‐ABL. Moreover, time course experiments using 2 µmol/L of ACF showed that reduction of STAT5A/5B phosphorylation and expression was detected after 48 hours of ACF treatment while activation of STAT3 was already maximal after 24 hours of treatment (Figure 3B). With respect to mRNAs levels, ACF dose‐dependently decreased the expression of *STAT5A*, *STAT5B*, but not *STAT3* (Figure 3C). Thus, STAT5A and STAT5B suppression might be related to an inhibitory effect of ACF on *STAT5A* and *STAT5B* transcription or mRNA stability. In addition, ACF dose‐dependently decreased the expression of known STAT5 target genes such as *PIM1*, *cMYC* and *CISH* (Figure 3D), keeping with a reduction of STAT5 activity.

We then addressed whether hypoxia regulates ACF‐mediated changes in STAT3/STAT5 phosphorylation and/or expression. K562 cells were cultured in normoxic (21% O_2_) or hypoxic (1% O_2_) conditions during ACF treatment. The actual establishment of hypoxic conditions was confirmed by the activation of HIF‐2α (Figure 3E). In hypoxia, like in normoxia, ACF reduced STAT5A and STAT5B expression and increased STAT3 phosphorylation (Figure 3E). This indicated that ACF oppositely regulates STAT3 and STAT5 signalling in K562 cells in a HIF‐independent manner.

**FIGURE 4:**
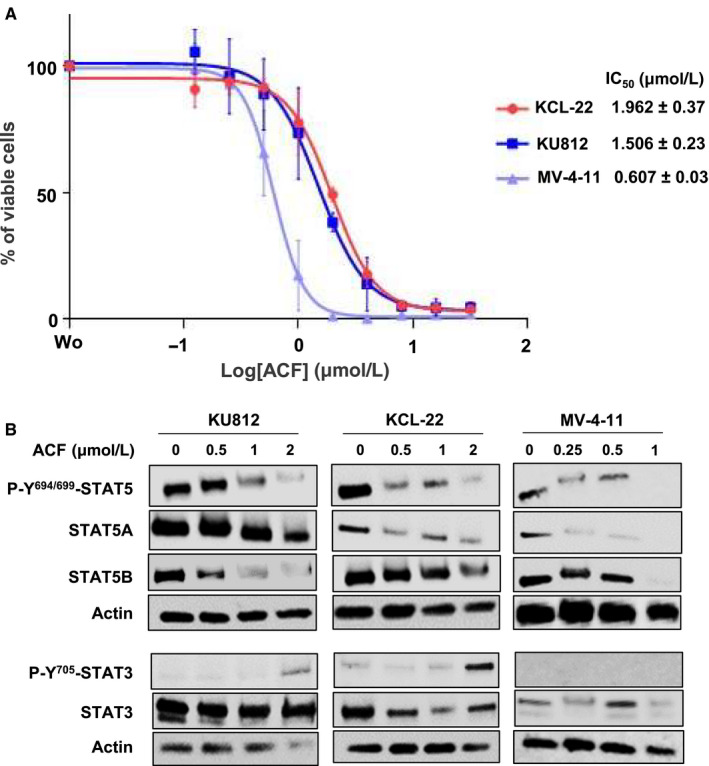
Acriflavine (ACF) inhibits cell growth and STAT3/5 signalling in various myeloid leukaemia cell lines. A, KU812, KCL‐22 and MV‐4‐11 cells were treated with various concentrations of ACF for 72 h, and the percentages of viable cells were determined by MTT assays (data are presented as mean ± SD of three independent experiments performed in triplicates). B, Protein extracts from K562, KCL‐22 and MV‐4‐11 cells cultured for 72 h without or with increasing concentrations of ACF were analysed by western blot to detect changes in phosphorylation and/or expression of STAT3/5A/5B (n = 2). Actin served as the loading control

To exclude the possibility that ACF effects on STAT3/5 signalling are a peculiarity of K562 cells, we tested the drug on the growth as well as on the activation and expression of STAT3/5 in two other BCR‐ABL‐positive cell lines (KU812 and KCL‐22) and in an AML cell line (MV‐4‐11) expressing FLT3‐ITD (Figure 4A). ACF inhibited the growth of all cell lines with IC_50_ values below 2 µmol/L with a maximal effect on the AML cell line. We then determined the impact of ACF on STAT3/STAT5 signalling and found that, like in K562 cells, ACF dose‐dependently activated STAT3, while suppressing STAT5A and STAT5B expression in KU812 and KCL‐22 cells. These effects were maximal in MV‐4‐11 cells. However, STAT3 phosphorylation in these cells, unlike in the BCR‐ABL‐positive cell lines, remained undetectable after ACF treatment (Figure 4B). Overall, these data suggest that ACF blocks STAT5A/5B expression in both CML and AML cells and that inhibition of cell growth induced by ACF may depend on STAT5 down‐regulation rather than STAT3 activation.

### 3.3 ACF overcomes the resistance of K562 cells to IM

**FIGURE 5:**
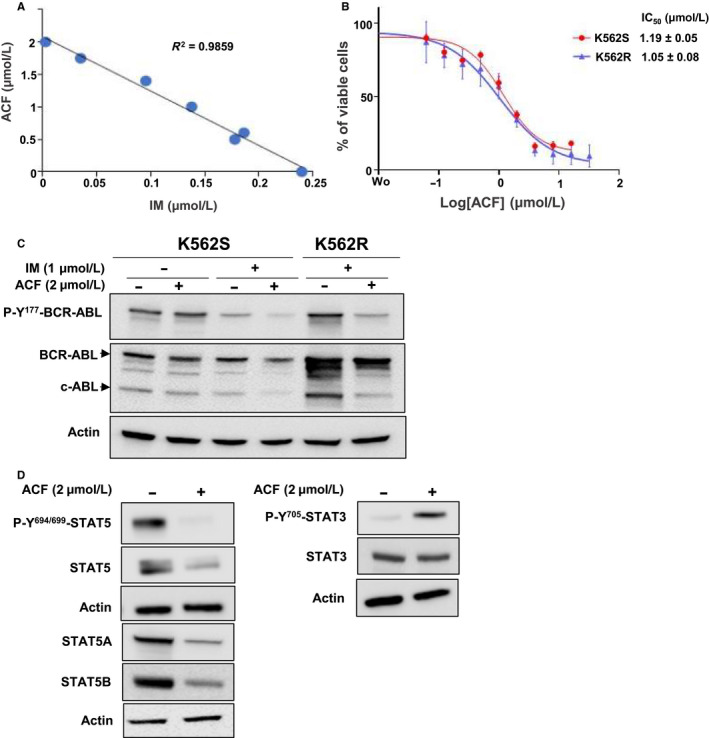
Combined effects of acriflavine (ACF) and imatinib (IM) on sensitive and resistant K562 cells. A, K562S cells were treated with different concentrations of ACF and IM for 72 h, and the percentages of viable cells were determined by MTT assays. Isobologram analysis was established according to values obtained with combined drugs in dose‐response experiments. B, K562S and K562R cells cultured, without and with 1 μmol/L IM, respectively, were treated with increasing concentrations of ACF for 72 h, and the percentages of viable cells were determined by MTT assays (data are presented as mean ± SD of four independent experiments done in triplicates). C, Protein extracts from K562S and K562R cells treated with ACF and/or IM for 72 h were analysed by western blot to detect the phosphorylation and expression of BCR‐ABL. D, The phosphorylation and expression of STAT3/5A/5B in K562R cells treated or not with ACF (2 μmol/L) were also determined by immunoblotting. C and D, Actin served as the loading control

We next analysed the effects of the combination of ACF with IM on IM‐sensitive K562 (K562S) and IM‐resistant K562 (K562R) cells (Figure 5). The isobologram showed that the combination of various concentrations of ACF and IM resulted in additive rather than synergistic effects on the growth of K562S cells (Figure 5A). In line with these results, K562S and K562R cells exhibited the same sensitivity to ACF (Figure 5B). When combined with IM, ACF slightly enhanced the reduction of BCR‐ABL phosphorylation and expression in both K562S and K562R cells (Figure 5C). Finally, the effects of ACF on STAT3 and STAT5 were confirmed in K562R cells (Figure 5D). ACF suppressed STAT5 phosphorylation and strongly inhibited STAT5A and STAT5B expression, whereas the phosphorylation of STAT3 was strongly stimulated, with no effect on its expression. Together, these results suggest that ACF might be a very useful drug that could be employed in combination with IM in order to eradicate resistant cells.

## 4 DISCUSSION

Targeted therapies are rapidly growing and became a reality for the treatment of cancers. Identification of key oncogenic drivers has led to the development of drugs that have improved the prognosis of cancer patients. The best example is the discovery of IM, the first TKI targeting BCR‐ABL and actually the best example of an antileukaemic compound that revolutionized the treatment of CML. However, IM is not totally curative with 50% of CML patients relapsing after IM discontinuation, pointing to the presence of IM‐resistant LSCs in the BM of CML patients.^35^ Mutations in the IM binding region of BCR‐ABL or activation of alternative signalling pathways mainly explained the acquired or de novo resistance to TKI.^36^ The BM microenvironment provides a sanctuary for LSCs. Hypoxia, a fundamental microenvironmental determinant, has been associated with the maintenance of CML LSCs through activation of HIFs in a BCR‐ABL‐independent manner.^16^ Similarly, activation of STAT3 or STAT5 via a JAK‐dependent but BCR‐ABL‐independent pathway promotes IM resistance of CML cells in BM microenvironment models.^28^, ^37^, ^38^ Moreover, STAT5 has been shown to play a key role in the maintenance of LSCs from CML patients, whereas combining IM with a STAT5 inhibitor triggers the death of CML LSCs.^21^ As a HIF inhibitor, ACF also targets CML LSCs while ACF combined with IM was more effective than IM alone in killing CML cells in low oxygen conditions.^18^ Targeting both HIF and STAT proteins might therefore help to eradicate CML LSCs and to prevent relapse after TKI combination therapy.

In this work, we demonstrated that ACF affects the growth and survival of CML cells by targeting STAT3 and STAT5 in hypoxic and normoxic conditions. We found that ACF dramatically reduced STAT5A and STAT5B expression and induced the tyrosine phosphorylation of STAT3 in CML cells. These changes were accompanied by cell growth arrest and apoptosis. The activation of STAT3 in ACF‐mediated inhibition of leukaemic cell growth remains however unclear. Chemotherapeutic agents such as doxorubicin have been previously shown to activate the JAK1/STAT3 pathway via the autocrine expression and secretion of IL‐6, suggesting that ACF might induce the tyrosine phosphorylation of STAT3 via this JAK kinase.^39^ However, the phosphorylation of STAT3 was not detected in MV‐4‐11 cells after ACF treatment. Moreover, we failed to observe any change in the ability of ACF to inhibit cell growth in the presence of STAT3 inhibitors (data not shown). Collectively, these data indicated that activation of STAT3 might be dispensable for ACF‐mediated growth inhibition of AML and CML cells. In fact, activation of STAT3 was demonstrated to be an important positive autocrine‐paracrine feedback loop in the therapeutic treatment of oncogene‐addicted cancer cells and might reflect a normal stress response in our experiments rather than a direct contribution of STAT3 in ACF‐mediated growth inhibition.^40^ ACF down‐regulates the expression of both forms of STAT5 at the protein and mRNAs levels, suggesting that ACF blocks *STAT5A/5B* gene expression. Interestingly, previous reports indicated that the PPARγ ligand, pioglitazone, also decrease *STAT5A/5B* mRNA levels in CML LSCs and reduced their clonogenic activity and long‐term potency in vitro. ^21^ Furthermore, the down‐regulation of STAT5 mRNAs and achievement of a major molecular response were observed in CML patients treated with IM and pioglitazone, indicating that targeting *STAT5* gene expression might eradicate CML LSCs in vivo.^21^ In line with these data, we recently reported that a STAT5 inhibitor associated with IM suppresses the growth of IM‐resistant CML cells by blocking STAT5B protein expression.^41^

Importantly, we showed that ACF‐mediated effects on STAT3/STAT5 occur via a BCR‐ABL‐independent mechanism. We found that ACF alone neither affects the phosphorylation nor the expression of this TKO in IM‐sensitive cells, even though minor, but significant, changes were observed when ACF was associated with IM. Moreover, we also observed that ACF does not affect the constitutive phosphorylation of the serine/threonine kinase AKT, a crucial downstream effector of BCR‐ABL, supporting our findings that suppression of STAT5 expression is responsible for ACF‐mediated inhibition of leukaemic cell growth.^42^ The effects of ACF were observed in normoxic as well as in hypoxic conditions indicating that HIFs are dispensable for the regulation of STAT3/STAT5 signalling in cells treated with ACF. Conversely, the suppression of STAT5 expression might directly interfere with HIF‐2α expression. Indeed, *HIF‐2α* was previously shown to be a target gene of STAT5 in CML LSCs and, in line, our data indicate that ACF inhibits the expression of both STAT5 and HIF‐2α proteins in K562 cells cultured in hypoxic conditions.^20^, ^21^ ACF is a multitarget drug that not only blocks HIF‐1α dimerization and STAT5 expression but also inhibits topoisomerases I/II and DNA‐PKcs.^2^, ^9^ These proteins play a critical role in controlling changes in DNA structure and/or DNA repair. Known inhibitors of these enzymes are employed as anticancer agents in the treatment of solid tumours and leukaemias. Most of them are DNA intercalators and induce accumulation of DNA double‐stranded breaks, cell death but also cellular senescence.^43^, ^44^ We also found that ACF, as an intercalating agent, induces DNA damage in CML cells at a concentration effective enough to favour apoptosis. At lower concentrations, ACF activates P21^Waf1^ protein which is recognized not only as a cyclin‐dependent kinase inhibitor but also as an important marker of cellular senescence suggesting that ACF might also induce a senescent phenotype in K562 cells.^45^

In summary, our data indicate that the simultaneous inhibition of HIFs and STAT5 might provide new therapeutic opportunities for relapsed CML. This can be achieved by combining different drugs or by employing multitarget inhibitors such as ACF. In addition, blocking both HIFs and STAT5 with ACF, that has been proven to be safe in humans, might help to reduce the side effects of combination therapies.

## CONFLICT OF INTEREST

The authors declare no conflict of interest.

## AUTHOR CONTRIBUTION

**Rawan Hallal:** Data curation (lead); investigation (lead); methodology (lead). **Rawan Nehme:** Data curation (equal); investigation (equal); methodology (equal). **Marie Brachet‐Botineau:** Data curation (supporting); investigation (supporting); methodology (supporting). **Ali Nehme:** Investigation (supporting); methodology (supporting). **Hassan Dakik:** Investigation (supporting); methodology (supporting). **Margaux Deynoux:** Investigation (supporting); methodology (supporting). **Persio Dello Sbarba:** Formal analysis (supporting); writing – review & editing (supporting). **Yves Levern:** Formal analysis (supporting); investigation (supporting). **Kazem Zibara:** Formal analysis (equal); supervision (supporting); writing – original draft (equal); writing – review & editing (equal). **Fabrice Gouilleux:** Conceptualization (lead); formal analysis (equal); supervision (lead); validation (equal); writing – original draft (lead). **Frédéric Mazurier:** Conceptualization (lead); formal analysis (lead); funding acquisition (lead); project administration (lead); supervision (lead); validation (lead); writing – original draft (lead).

## Data Availability

The data that support the findings of this study are available from the corresponding author upon reasonable request.
